# Evaluating the impacts of sustainable land management practices on water quality in an agricultural catchment in Lower Austria using SWAT

**DOI:** 10.1007/s10661-023-11079-y

**Published:** 2023-03-25

**Authors:** Francis Kilundu Musyoka, Peter Strauss, Guangju Zhao, Stefan Strohmeier, Benedict Mwavu Mutua, Andreas Klik

**Affiliations:** 1 Department of Water, Atmosphere and Environment, University of Natural Resources and Life Sciences, Vienna, Institute of Soil Physics and Rural Water Management, Muthgasse 18, 1190 Vienna, Austria; 2 Institute for Land and Water Management Research, 3252 Petzenkirchen, Austria; 3 Institute of Soil and Water Conservation, Northwest A & F University, Yangling, Shaanxi 712100 China; 4 Division of Planning, Partnerships, Research and Innovation, Kibabii University, Bungoma, Kenya

**Keywords:** Soil conservation, Agricultural management practices, SWATplusR, Nonpoint source pollution, Hydrological modeling, Catchment hydrology

## Abstract

Managing agricultural watersheds in an environmentally friendly manner necessitate the strategic implementation of well-targeted sustainable land management (SLM) practices that limit soil and nonpoint source pollution losses and translocation. Watershed-scale SLM-scenario modeling has the potential to identify efficient and effective management strategies from the field to the integrated landscape level. In a case study targeting a 66-hectare watershed in Petzenkirchen, Lower Austria, the Soil and Water Assessment Tool (SWAT) was utilized to evaluate a variety of locally adoptable SLM practices. SWAT was calibrated and validated (monthly) at the catchment outlet for flow, sediment, nitrate-nitrogen (NO_3_–N), ammonium nitrogen (NH_4_–N), and mineralized phosphorus (PO_4_–P) using SWATplusR. Considering the locally existing agricultural practices and socioeconomic and environmental factors of the research area, four conservation practices were evaluated: baseline scenario, contour farming (CF), winter cover crops (CC), and a combination of no-till and cover crops (NT + CC). The NT + CC SLM practice was found to be the most effective soil conservation practice in reducing soil loss by around 80%, whereas CF obtained the best results for decreasing the nutrient loads of NO_3_–N and PO_4_–P by 11% and 35%, respectively. The findings of this study imply that the setup SWAT model can serve the context-specific performance assessment and eventual promotion of SLM interventions that mitigate on-site land degradation and the consequential off-site environmental pollution resulting from agricultural nonpoint sources.

**Supplementary Information:**

The online version contains supplementary material available at 10.1007/s10661-023-11079-y.

## Introduction

Nonpoint source pollution can cause substantial harm to the environment and counteract sustainable watershed management attempts (Chaubey et al., 2010). Intensive agricultural practices lead to excessive quantities of sediments and nutrients in the surrounding water bodies (Novotná et al., 2014; Strauss et al., 2007). Soil erosion and excessive amounts of nitrogen and phosphorus in the environment are primarily related to surface runoff and land drainage. They are critical contributors to environmental degradation leading to water quality deterioration in watersheds (Özcan et al., 2017; Schönenberger et al., 2022; Tuppad et al., 2010). Additionally, the depletion of topsoil and vital nutrients from farmlands lowers land productivity and increases the intensity of agricultural management (Wagena & Easton, 2018).

To address the agricultural pollution challenge, the European Union (EU) adopted the Water Framework Directive (European Commission, 2012) to establish a legislative framework for water quality and quantity in Europe (Abbaspour et al., 2015; Malagó et al., 2017). Additionally, the Nitrates Directive aims to restrict and avoid future water contamination resulting from nitrates emanating from agricultural activities (Malagó et al., 2017). The Commission’s mandate is to act and reduce nitrogen and phosphorus losses by at least 50% while preserving the productivity and fertility of agricultural soils. The directive also aims to reduce the use of fertilizers in agriculture by at least 20% by 2030 (European Commission, 2020).

Implementing a system of agricultural conservation practices, otherwise known as sustainable land management (SLM) practices at farm level, is a viable approach for minimizing nutrient and sediment pollution from agricultural watersheds (Arabi et al., 2008). SLM practices are described as techniques for soil and water conservation as well as social actions developed for specific agricultural lands that strive towards sustainability while representing realistic and practical environmental preservation measures (Laurent & Ruelland, 2011; Özcan et al., 2017). A single agricultural technique rarely solves a pollution problem; instead, a combination of approaches should be applied (Chiang et al*.*, 2014). It is usually up to the authorities and the farmers to determine which SLM practice combinations are well-suited for achieving a better balance between agricultural productivity and environmental conservation (Sharpley et al., 2006).

Assessment of management practices at a watershed scale through field campaigns is costly and time-consuming (Lamba et al., 2016; Santhi et al., 2006). For this reason, evaluating the impacts of agricultural management practices on water quality at the catchment scale necessitates the use of cost-effective and time-efficient modeling tools that integrate both agricultural and hydrological aspects within the watershed (Neitsch et al., 2011; Ni & Parajuli, 2018). Many hydrologic models have been developed over the years (Lam et al., 2011). Some of the commonly used models are the Areal Nonpoint Source Watershed Environment Response Simulation (Beasley et al., 1980), MIKE SHE (Jaber & Shukla, 2012), Agricultural Non-Point Source Pollution System (Bingner et al., 2018), Hydrological Simulation Program-Fortran (Duda et al., 2012), and Soil and Water Assessment Tool (SWAT) (Neitsch et al., 2011). Among these models, SWAT provides the broadest range of agricultural management alternatives (Kalin & Hantush, 2003) and is therefore very well suited for evaluating multiple impacts of SLM practices on hydrology, nutrients, and soil loss.

Due to its flexible framework, SWAT is widely used (Lam et al., 2011; Lamba et al., 2016; Mtibaa et al., 2018; Sharpley et al., 2006; Strauch et al., 2013) to evaluate the effect of land management and agricultural practices on water quality (Liu et al., 2013). Zhao et al. (2001), Tuppad et al. (2010), and Salm and Chardon (2007) applied SWAT model in watersheds of varying sizes and characteristics in different geographical locations and reported a reduction in sediment and phosphorus loads after agricultural management practices including conservation tillage, contour farming, and fertilizer management had been implemented. Lam et al. (2011) reported a 9 to 20% decrease in annual nitrate load in a lowland catchment in Germany after implementation of best management practices.

Even though agricultural management practices have been extensively modeled with SWAT, most studies have focused on sediment and runoff yields. Due to the challenges involved in obtaining good nutrients data, the research on the impact of land management practices on nutrient loading has been based on non-point source nutrient estimates or regional data (Rousseau et al., 2012). The availability of high-quality nutrients data in this research could therefore be a valuable addition into the nutrients research and modeling. The objective of this study was to utilize the SWAT model to evaluate the impact of selected SLM practices, including the current practices implemented in the catchment, refered in this paper as the baseline scenario (BS), contour farming (CF), cover crops during the winter period (CC), and combined no-till and cover crops (NT + CC) in a 66-ha agricultural watershed on runoff, erosion processes, and nutrient losses. The investigated catchment is heavily monitored and therefore, extensive, high-quality data on hydrology, climate, and agricultural land management is available. To achieve the objectives, the model’s performance in simulating runoff, nitrate-nitrogen, ammonium nitrogen, mineralized phosphorus, and sediments was assessed through the calibration and validation procedure. Following a satisfactory model performance, the selected SLM practices were simulated, and their potential impacts on water quality and quantity were evaluated.

## Materials and methods

### Study area

**Fig. 1:**
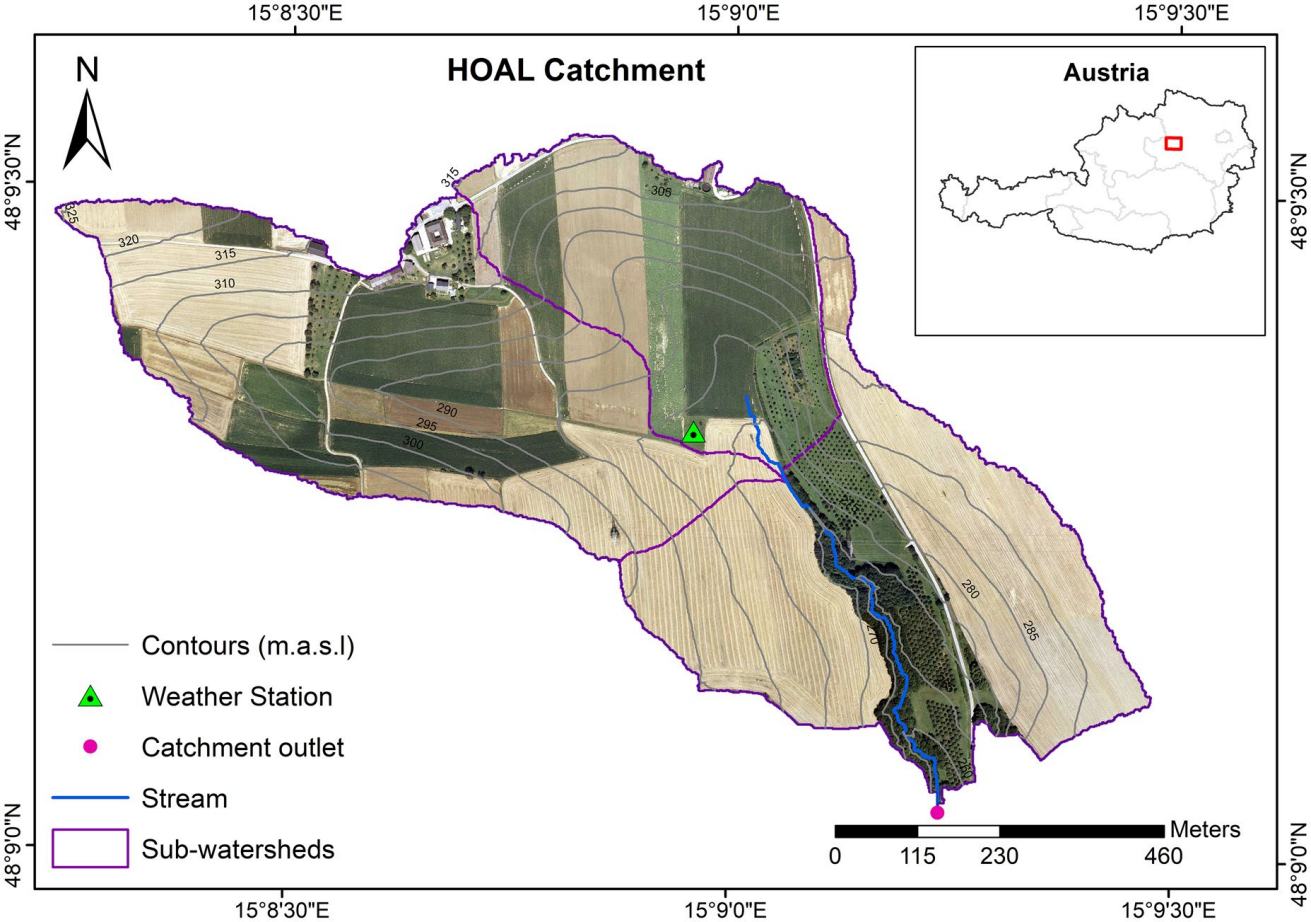
The study area: HOAL catchment with SWAT-defined sub-watersheds and monitoring stations

This study was carried out at the Hydrological Open Air Laboratory (HOAL) catchment located in Petzenkirchen (48° 9′ N, 15° 9′ E), Lower Austria, roughly 100 km west of Vienna (Blöschl et al., 2016). The watershed is 66 ha in size, and its elevation ranges from 257 to 323 m.a.s.l, with an average slope of 8% (Picciafuoco et al., 2019). Agriculture is the most common land use in the area, accounting for 87% of the total catchment area (Fig. 1). The remaining 13% of the watershed’s surface area consists of meadows and paved areas (Musyoka et al., 2021). Based on data from 2000 to 2018, the annual average precipitation is 819 mm, while the annual average temperature is 9.3 °C. During summer, air temperature and rainfall volume reach seasonal highs whereas the catchment receives very little snow throughout the winter, which evaporates quite fast (Széles et al., 2020). The geology of the HOAL catchment is composed of tertiary fine sediments and molasse zone fractured siltstone (Blöschl et al., 2016). Due to the fragile nature of the soil’s parent material (Tertiary fine sediments and fractured siltstone of the Molasse zone), the catchment is prone to soil erosion when intensive farm management activities are carried out in the agricultural fields (Strauss et al., 2007). The most widespread soil types are Cambisols (57%), Planosols (21%), and Kolluvisols (16%), all of which have medium to poor permeability (Széles et al., 2020). The principal crops grown in the watershed are maize, winter barley, winter wheat, and rapeseed. Crop rotation is used in conjunction with mineral fertilization and partially with farmyard manure to provide natural soil fertilization. The cropland is divided into thirty-seven fields, each with its agricultural management system and fields range in size from 1 to 12 ha, with an average field area of 1.5 ha (Musyoka et al., 2021).

### Soil and water assessment tool description

SWAT is a physically based continuous watershed model developed to simulate the effects of various land management practices on water, sediment, and other agricultural flows (Arnold et al., 1998). The model is commonly applied in large watersheds with varying land uses, soils, and management scenarios. However, the model has also been used to simulate hydrological processes in small catchments (Govender & Everson, 2005; Green & van Griensven, 2008; Kannan et al., 2006, 2007; Roth & Lemann, 2016). Hydrology, erosion, plant development, weather, nutrients, pesticides, soil temperature, and agricultural management are the key components of the model (Green & van Griensven, 2008). The water balance equation (Neitsch et al., 2002), presented in Eq. (1), simulates the hydrologic cycle

1 $${SW}_{t}={SW}_{o}+\sum_{i=1}^{t}\left({R}_{day}-{Q}_{surf}-{E}_{a}-{W}_{seep}-{Q}_{gw}\right)$$

where *SW*_*t*_ is the final soil water content (mm), *SW*_*o*_ is the initial soil water content on day *i* (mm), *t* is the time (day), *R*_*day*_ is the amount of precipitation on day *i* (mm), *Q*_*surf*_ is the amount of surface runoff on day *i* (mm), *E*_*a*_ is the amount of evapotranspiration on day *i* (mm), *W*_*seep*_ is the amount of water entering the vadose zone from the soil profile on day *i* (mm), and *Q*_*gw*_ is the amount of return flow on day *i* (mm).

To account for geographical diversity, the model divides the watershed into a number of sub-watersheds depending on the topography and critical source area (Kumar et al., 2014). Based on unique combinations of soil, slope, and land use types, each sub-watershed is subsequently divided into hydrologic response units (HRUs) (Tuppad et al., 2010). HRUs are the smallest SWAT spatial features used to compute water balance components (Yao et al., 2016). Surface runoff, soil moisture, sediment output, crop development, nutrient cycles, and agricultural management practices are simulated at HRU level before being averaged for the sub-basin (Tuppad et al., 2010). The averaged sub-basin values are then routed to the outlet through channels, wetlands, and reservoirs (Teshager et al., 2016). The SWAT hydrological computation is divided into two phases: the land phase, where the model calculates the upland loadings of discharge, soil loss, and nutrients from each HRU, which are then area-weighted to the sub-basin level; and the routing phase, where loadings are routed from each sub-watershed via the stream network (Zhu & Li, 2014). The overland flow velocity is influenced by the land surface characteristics and is estimated in SWAT by the Manning’s roughness coefficient for overland flow (OV_N) parameter (Neitsch et al., 2011).

The modified universal soil loss equation (MUSLE) (Williams & Berndt, 1977) estimates erosion at the HRU and sub-basin levels (Engebretsen et al., 2019), while surface runoff is calculated by the modified Soil Conservation Service-Curve Number method (Ricci et al., 2020). Furthermore, a modified Bagnold’s equation is used to model in-stream sediment transport (Neitsch et al., 2002). The MUSLE equation can be expressed as shown in Eq. (2).

2 $${S}_{ed}=11.8{\left({Q}_{surf}\cdot {q}_{peak}\cdot {area}_{hru}\right)}^{0.56}\cdot {K}_{USLE}\cdot {C}_{USLE}\cdot {P}_{USLE}{\cdot LS}_{USLE}\cdot CFRG$$

where *S*_*ed*_ is the sediment yield (tons), *Q*_*surf*_ is the surface runoff volume (mm/ha), *q*_*peak*_ is the peak runoff rate (m^3^/s), *area*_*hru*_ is the area of the HRU (ha), *K*_*ULSE*_ is the USLE soil erodibility factor, *C*_*ULSE*_ is the USLE cover and management factor, *P*_*ULSE*_ is the USLE support practice factor, *LS*_*USLE*_ is the USLE topographic factor, and *CFRG* is the coarse fragment factor.

Nitrogen is a highly reactive element that can exist in various dynamic forms and phases, including nitrate, nitrite, and ammonium in water and soil (Zettam et al., 2020). Five different pools of nitrogen are monitored within SWAT (Neitsch et al., 2011), where three of the pools consist of organic forms of nitrogen, and the other two pools are the inorganic forms of nitrogen (NO_3_^−^ and NH_4_^+^) (Lam et al., 2011). Nitrate is computed as a product of soil layer nitrate concentration and runoff volume while sediment yield in a given day, phosphorus enrichment ratio, and phosphorus concentration attached to the top 10 mm of soil are used to estimate phosphorus attached to sediment (Giri et al., 2012). The proportion of soluble phosphorus, on the other hand, is determined by the concentration of phosphorus in the solution, the volume of runoff, and the partitioning factor (Neitsch et al., 2011).

The digital elevation model (DEM), daily weather data, land use/land cover, and soil types are the model’s main inputs necessary to simulate hydrological processes in a catchment (Mengistu et al., 2019). A DEM with a resolution of 1 m was utilized in this study while maps, field observations, and farm records were used to develop the land use map. Complete information on the agricultural land management operations (2008–2018) including planting and harvesting dates, date and type of tillage implements used, and type, date, and amount of fertilizer applied was available for each of the 37 fields (Musyoka et al., 2021). Nitrogen application rates through inorganic fertilizers vary for each agricultural field. Nevertheless, the mean annual nitrogen application rate per field (2008–2018) is 121 kg per hectare, while that of phosphorus is 62 kg per hectare per year. Liquid manure mainly consisting of pig slurry is also applied in some agricultural fields at a rate of around 20 m^3^/ha per year. Soil map was developed from the measured soil physical and chemical properties based on the procedure described by Musyoka et al. (2021). Table 1 provides a summary of the availability and resolution of the input data.

**Table 1:** Input data sources and resolution

**Data**		**Resolution/description**
Input data	DEM	1 m × 1 m
	Soils map	5 m × 5 m
	Land use map	5 m × 5 m
	Climate data (precipitation, temperature (minimum and maximum), relative humidity, wind velocity, solar radiation)	Minute interval
	Management information	Planting, harvest, type of implement, tillage, and fertilizer application dates and rates
Model calibration and validation data	Discharge, sediment yield, soil moisture	Minute interval
	NO_3_–N, NH_4_–N, and PO_4_–P	Grab samples

Data source: Federal Agency for Water Management

Nitrates, phosphorus, and ammonium samples are not collected on a regular basis, but instead, samples are taken in at least 10 days each month. To compare the simulated and observed nutrient concentrations, only the measured daily values were converted to monthly sums and compared with the same days from the SWAT simulation results. These monthly totals were assumed to be representative of the monthly nutrient loads in the HOAL catchment

The relationship between water discharge and nutrient concentration is highly variable and can be influenced by many factors (D’Amario et al., 2021). To calculate the monthly nutrient loads, the concentration of the nutrients for each measurement was multiplied by the corresponding flow to obtain the nutrient loading per day. The loadings were then summed up to obtain monthly nutrient loadings. The monthly loads were calculated using the formula below adapted from Zettam et al. (2020).

3 $$F=\sum_{d=1}^{n}\left({Q}_{d}{C}_{d}\right)$$

where *F* is the monthly nutrient loading (kg), *Q*_*d*_ is the flow on day *d* (m^3^/s), *C*_*d*_ is the nutrient concentration in the stream (mg/l), and *n* is the number of days in the month when the measurements were taken: model setup, sensitivity analysis, calibration, validation, and evaluation.

The automated delineation tool included in ArcSWAT 2012 was used to define sub-basins and streams. The catchment was subdivided into three sub-watersheds and 212 HRUs. Further information on land use, agricultural management practices, and evapotranspiration methods are outlined by Musyoka et al. (2021).

Model calibration necessitates the identification of parameters that are more sensitive to value changes, whereby a minimal change in the parameter generates a substantial change in the result. Modelers can utilize sensitivity analysis to determine which parameters most significantly contribute to output variance due to input variability. This makes it possible to select only the most crucial parameters and reduces the parameters that need parameterization (Holvoet et al., 2005; Perez-Valdivia et al., 2017).

Sensitivity analysis was conducted through the Fourier Amplitude Sensitivity Test (FAST) (Cukier et al., 1973), a variance-based technique for model sensitivity analysis (Musyoka et al., 2021). FAST decomposes the variance of model results into the partial variances given by model parameters using a systematic sampling approach and a Fourier transformation. The partial variance to model output variance ratio is used to quantify the significance of parameters in terms of their contribution to model output uncertainty (Xu & Gertner, 2008). The sensitivity index provides a measure of how sensitive a system is to changes in its parameters, which can be used to identify the critical parameters (Saltelli et al., 1999). FAST is a computationally efficient approach and has been used to evaluate the sensitivity of soil erosion and hydrological models (Francos et al., 2003; Guse et al., 2014). The sensitivity analysis for the SWAT model was conducted with the help of the R package FAST (Reusser, 2015).

Based on the available water quality monitoring information, ammonium nitrogen (NH_4_–N), mineralized phosphorus (PO_4_–P), and nitrate-nitrogen (NO_3_–N) were used to assess the water quality in the catchment. Initially, SWAT parameters affecting surface runoff response, soil loss, NO_3_–N, NH_4_–N, and PO_4_–P were selected to investigate model sensitivity. The model parameters were subjected to sensitivity analysis using their default values. Afterward, the model was calibrated using the identified sensitive parameters.

Discharge, sediment, NO_3_–N, PO_4_–P, and NH_4_–N were all calibrated on a monthly time step. Daily calibration for flow, soil erosion, and soil moisture content for the HOAL catchment has done previously using the SWAT model, achieving good results (Musyoka et al., 2021). Even though flow and sediment data were available on a sub-daily time step, nutrient data was available only on some specific days of the month. Therefore, all the components were calibrated at a monthly interval for uniformity. A 2-year period (2010–2011) was used as warm-up period. Moriasi et al. (2007) recommend 3 to 5 years of data for optimal model calibration and validation. Calibration was therefore performed for the period 2012 through 2015, while the model validation for the same components was carried out from 2016 to 2018.

### Model evaluation

The relationship between model predicted and observed data was evaluated through graphical and statistical approaches. The R package SWATplusR (Schürz, 2019) was used for the evaluation of the model performance. SWATplusR contains tools that integrate the SWAT output into R, allowing users to perform simulations as well as regulating modifications in model parameters, simulation periods, and time intervals (Musyoka et al., 2021; Schürz, 2019).

The coefficient of determination (*R*^2^), the Nash–Sutcliffe Efficiency (NSE) (Nash & Sutcliffe, 1970), the Percent Bias (PBIAS), and the root mean square error (RMSE) were used to assess the fit between the simulated and measured data. NSE values can vary from 1 to negative 1. NSE value of 1 indicates a perfect match between simulated and observed data, while values between 0 and 1 represent the most optimal parameters for model performance (Baker & Miller, 2013). The coefficient of determination expresses the degree of agreement between measured and simulated data (Wen et al., 2013), whereas PBIAS expresses the propensity of predicted data to be larger or less than the measured data (Moriasi et al., 2015).

### Sustainable land management practices implementation in SWAT

**Fig. 2:**
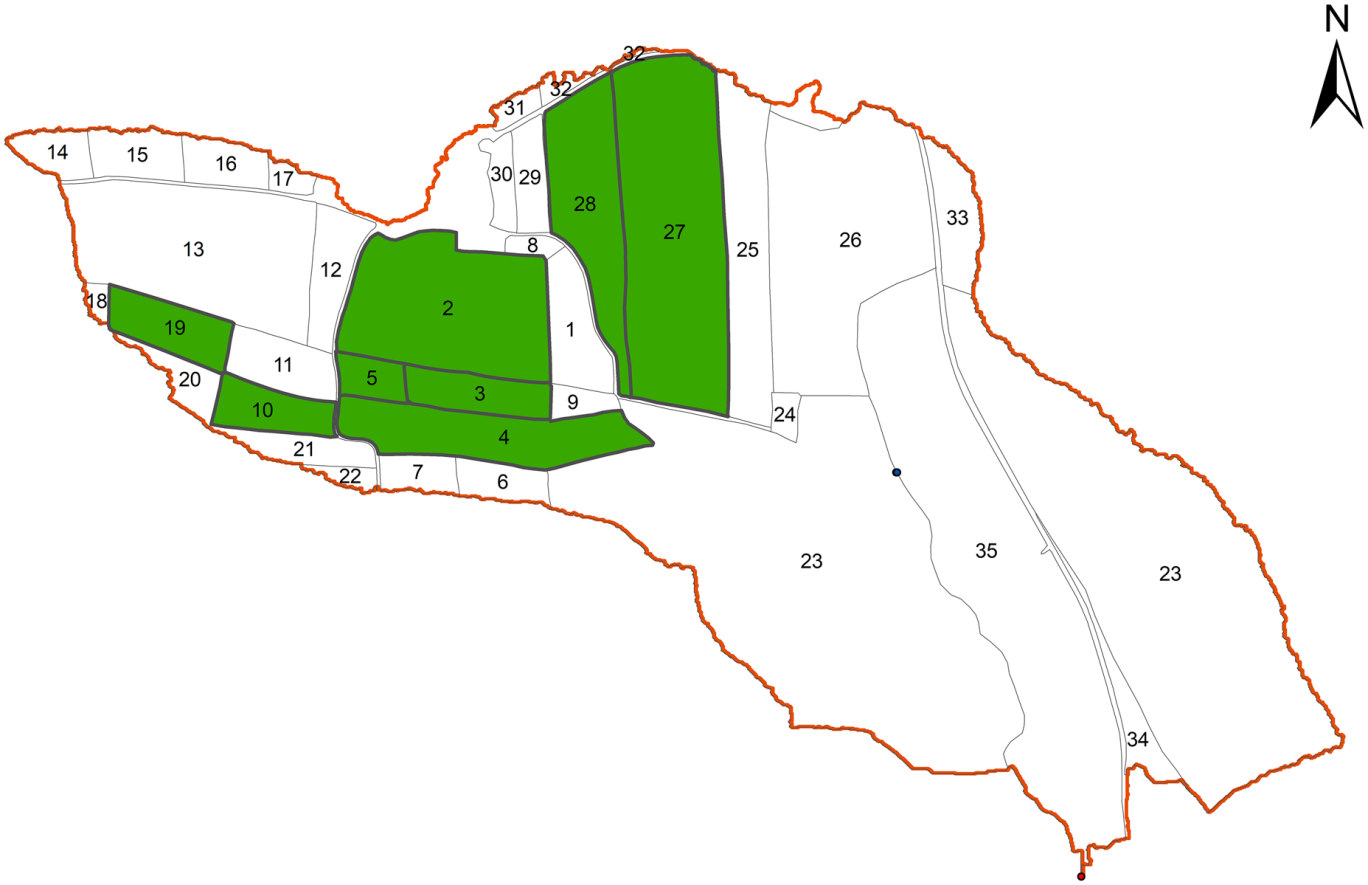
Agricultural fields in the HOAL catchment where winter cover crop practice has been implemented (green plots)

The simulated SLM practices included four management practices: (1) BS, (2) CF, (3) CC, and (4) a NT + CC. The current management practices in the catchment revolve around corn and winter wheat crop rotation. Occasionally, winter barley, soybeans, sunflower, and rapeseed are cultivated in some fields. Depending on the season, maize is commonly planted in late April or early May and harvested around October. Winter wheat is sown in late October or early November and harvested in July of the following year (Table 2). Cover crops are planted in mid-August after harvesting winter wheat or corn and removed towards the end of November or early December in some of the agricultural fields, while some other fields are left bare until the next planting. A mixture of cover crops, including phacelia, mustard, clovers, canola, and alfalfa, are grown as cover crops in some fields in the catchment as shown in Fig. 2. The current management practice was simulated under the BS.

**Table 2:** Example of a two-year (2012–2013) agricultural management practices for the SLM practices under consideration

**Date**	**Management operation**	**CF/baseline**	**CC**	**NT + CC**
20/03/2012	Fertilizer application	NAC (275 kg/ha)		
10/5/2012	Fertilizer application	NAC (195 kg/ha)		
25/5/2012	Fertilizer application	NAC (208 kg/ha)		
23/7/2012	Harvest winter wheat	Harvester		
30/7/2012	Tillage	Field cultivator	Field cultivator	Generic no-till
13/08/2012	Planting clovers	Direct seeding		
15/11/2012	Kill clovers	Mouldboard plough	–	–
30/3/2013	Kill clovers	–	Mouldboard plough	Generic no-till
16/4/2013	Tillage	Field cultivator	Field cultivator	–
23/4/2013	Fertilizer application	NAC (470 kg/ha)		
27/4/2013	Tillage	Field cultivator	Field cultivator	Generic no-till
27/4/2013	Planting corn	Corn single space drill		
13/10/2013	Harvesting corn	Harvester		
16/10/2013	Tillage	Mouldboard plough	Mouldboard plough	Generic no-till
19/10/2013	Planting winter wheat	Direct seeding with fore tools	Direct seeding with fore tools	Direct seeding without fore tools

*NAC* N-acetyl cysteine

Contour farming entails carrying out field operations such as cultivation, seeding, and harvesting along the contour (Liu et al., 2013). The main aim of contouring is to intercept runoff and prevent sheet and gully erosion by creating micro-contour ridges along the plough line, reducing the flow velocity of runoff and thereby limiting rill development, fostering water infiltration, and protecting gentle agricultural fields against storm erosion (Arabi et al., 2008; Liu et al., 2013; Tuppad et al., 2010). No-till causes less residue disturbance than the conventional tillage system, where deeper and more frequent ploughing is practiced (Lam et al., 2011). Unlike conventional tillage, no-till practices can reduce soil erosion by water and increase soil water infiltration rate by leaving plant material on the ground and root systems undamaged and macro-pore systems connected (van Wie et al., 2013). No-till practice, therefore, aids in water retention in the soil until the subsequent soil management. After-harvest cover crops provide comparable benefits to no-till farming, such as canopy formation and soil stabilization. Cover crops can fix nitrogen into the soil and enrich the soil with organic matter since they are not harvested but killed and integrated into the soil prior to the planting of the main crop (Arabi et al., 2008; Lam et al., 2011).

No-till farming was simulated through the generic no-till option provided by the SWAT database, reducing the tillage depth (DEPTIL) to 25 mm. Additionally, the mixing efficiency (EFTMIX) was decreased from 0.3 (conventional tillage) to 0.05 (no-till). The CN2 parameter was reduced by three units, as recommended by Waidler et al. (2011), while Manning’s roughness value for overland flow (OV_N) was increased from the default 0.15 to 0.3 (Neitsch et al., 2011). The C_USLE parameter was also decreased from the default 0.2 to 0.1 for row crops (corn, sunflower, soybeans, and canola) as suggested by Maski et al. (2008). Contour tillage was implemented by reducing the curve number by three units (Arabi et al., 2008) and the P-factor to 0.6 (Wischmeier & Smith, 1978). Cover crops were represented by clovers in the SWAT model.

### SLM practice evaluation

The SLM practices were evaluated in comparison to the baseline scenario. The effects of SLM practice implementation on sediment and water quantity and quality are shown as percentage changes in mean seasonal and annual discharge, sediment, nitrates, and phosphorus loadings at the watershed level. The loadings were calculated for each HRU and then totaled for the entire watershed. The change at the watershed level reflects overland load reductions from the HRUs as a result of SLM practice implementation. Except for the parameters used to represent an SLM practice, all inputs were kept constant during the evaluation process.

The Wilcoxon statistical test (Wilcoxon, 1945) was conducted on an annual time step to determine the statistical significance of each SLM practice. In total, 24 samples were used to run the Wilcoxon test. The test was selected because it is suitable for small sample sizes and applicability with paired or matched data (Gibbons & Chakraborti, 2020). The Wilcoxon statistical test is widely used to ascertain the statistical significance of paired data, irrespective of whether the data originated from the same source (Motsinger et al., 2016). To determine the significance of change as a result of SLM practices’ implementation, the results of the simulated SLM practices were compared to the calibrated model to evaluate any significant changes in flow, sediment yield, nitrate-nitrogen, and phosphorus. The SLM practice impact was deemed insignificant if the *p*-value was higher than 0.05.

## Results and discussion

### Sensitivity analysis

**Fig. 3:**
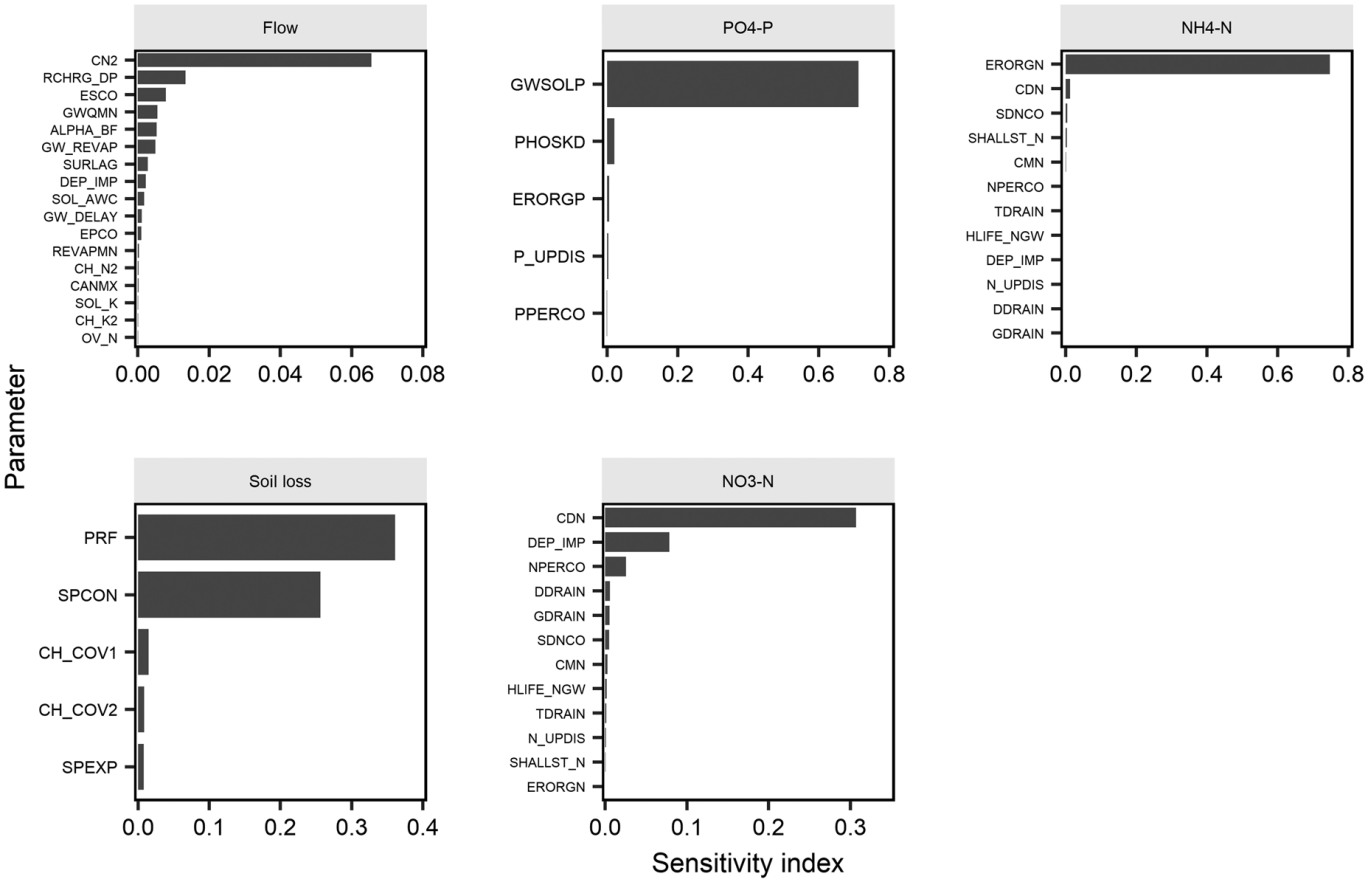
Model sensitivity results for flow, sediment, NO_3_–N, PO_4_–P, and NH_4_–N. The parameters are described in Table 3

Sensitivity analysis was conducted on the HOAL catchment flow, sediment, NO_3_–N, NH_4_–N, and PO_4_–P to establish the most sensitive SWAT parameters, which would be later used for model calibration and validation. Figure 3 shows the sensitivity analysis results for flow, sediment, NO_3_–N, NH_4_–N, and PO_4_–P. Sensitivity analysis (Fig. 3) revealed that the flow was primarily influenced by the curve number (CN2), while the sediment, PO_4_–P, NH_4_–N, and NO_3_–N were mainly affected by the peak rate adjustment factor for sediment routing in the main channel (PRF), the concentration of soluble phosphorus in groundwater contributing to streamflow from the sub-basin (GWSOLP), the organic N enrichment ratio for loading with sediment (ERORGN), and the denitrification exponential rate coefficient (CDN), respectively. In total, twenty-two parameters (Table 3) were identified as the most sensitive parameters for all components evaluated and were hence used for model calibration and validation. Previous studies have also identified these parameters as sensitive parameters for flow (Arabi et al., 2008; Gharibdousti et al., 2019; Storm et al., 2006), sediment (Yesuf et al., 2015), phosphorus (Tuppad et al., 2010), and nitrogen (Lam et al., 2011; Malagó et al., 2017; Zettam et al., 2020).

**Table 3:** SWAT parameters, their description, and ranges as used during model calibration and validation

**Parameters**	**Description**	**Model range**	**Value used**
**Flow**			
CN2^p^	Initial SCS runoff curve number for moisture condition II	−20 to 20	−5
ESCO^a^	soil evaporation compensation factor	0 to 1	0.80
GWQMN^a^	Threshold depth of water in the shallow aquifer required for return flow to occur (mm)	0 to 5000	500
EPCO^a^	Plant evaporation compensation factor	0 to 1	0.5
CH_K2^a^	Effective hydraulic conductivity in the main channel alluvium (mmh^−1^)	−0.001 to 500	40
ALPHA_BF^a^	Base flow recession factor	0 to 1	0.5
GW_REVAP^a^	Groundwater revap coefficient	0.02 to 0.2	0.15
RCHRG_DP^a^	Deep aquifer percolation fraction	0 to 1	0.48
SOL_AWC^p^	Available water capacity in the soil layer	−30 to 30	14
SURLAG^a^	Surface runoff lag time in the HRU (days)	0 to 24	2
GW_DELAY	Groundwater delay time (days)	0 to 500	

**Sediment**			
SPCON^a^	Linear parameter for calculating the maximum amount of sediment that can be reentrained during channel sediment routing	0.0001 to 0.01	0.006
PRF^a^	Peak rate adjustment factor for sediment routing	0 to 2	0.51
SPEXP^a^	Exponent parameter for calculating sediment reentrained in the channel sediment routing	1 to 1.5	1.5

**Nitrogen**			
CDN^a^	Denitrification exponential rate	0 to 3	0.009
CMN^a^	Rate factor for humus mineralization of active organic Nitrogen	0.001 to 0.003	0.002
NPERCO^a^	Nitrogen percolation coefficient	0 to 1	0.70
ERORGN^a^	Organic nitrogen enrichment ratio	0 to 1	0.13
SDNCO^a^	Denitrification threshold water content	0 to 1	0.90
SHALLST_N^a^	Nitrate concentration in shallow aquifer (mm)	0 to 1000	530

**Phosphorus**			
GWSOLP^a^	Soluble P concentration in groundwater loading (mg P/l)	0 to 1000	50
PHOSKD^a^	Phosphorus soil partitioning coefficient	100 to 200	175

^a^Absolute change, ^p^percent change

### Model calibration and validation

Monthly calibration and validation were conducted in the HOAL catchment outlet. For the model calibration, two iterations each consisting of 500 simulation runs were made using SWATplusR. The model parameters were adjusted and optimized based on the NSE values obtained after the first iteration. After calibration, the final range of parameters was used for model validation. Table 5 presents the outcome of model calibration and validation conducted for the monthly observed and simulated components at the watershed outlet. A water b based on the calibration results, the SWAT model satisfactorily predicted monthly stream flow patterns between 2012 and 2015. The monthly average flow predicted by the model was 3.6 l/s, which was more or less similar to the measured flow of 3.7 l/s for the 4-year calibration period. The model performed very well for flow according to the evaluation criteria proposed by Moriasi et al. (2007) (Table 4). The *R*^2^, NSE, PBIAS, and RMSE values were within the acceptable range for all the calibrated components, as shown in Table 5. The model performance during validation was also satisfactory for flow, sediment, PO_4_–P, and NO_3_–N. The NSE value for NH_4_–N validation, however, showed unsatisfactory results.

**Table 4:** General performance ratings for statistics based on Moriasi et al. (2007)

**Performance rating**	***R***^**2**^	**NSE**	**PBIAS**
Very good	> 0.75	> 0.75	< ± 10%
Good	0.65 < NSE ≤ 0.75	0.65 < NSE ≤ 0.75	± 10% ≤ PBIAS ≤ ± 15%
Satisfactory	> 0.5	≥ 0.5	± 15% ≤ PBIAS ≤ ± 25%
Unsatisfactory	≤ 0.5	< 0.5	> ± 25%
Acceptable	> 0.5	0 < NSE ≤ 1	< ± 50%
Unacceptable	≤ 0.5	< 0	> ± 50

**Table 5:** Long-term mean monthly measured data, model calibration (2012–2015) and validation results (2016–2018), and corresponding statistical comparisons

**Component**	**Mean**		**Std. dev**		**Median**		**NSE**	***R***^**2**^	**PBIAS**	**RMSE**
	Obs.	Sim.	Obs.	Sim.	Obs.	Sim.				
**Calibration**										
Flow (l/s)	3.73	3.67	3.32	3.37	2.96	2.69	0.86	0.86	−1.8	1.24
Sediment (t/mon)	0.34	0.34	0.57	0.48	0.08	0.11	0.79	0.79	−0.20	0.26
NO_3_–N (kg/mon)	13.25	12.35	19.51	19.20	6.74	5.64	0.76	0.77	−6.80	9.50
NH_4_–N (kg/mon)	0.06	0.06	0.07	0.09	0.04	0.03	0.47	0.63	−5.90	0.05
PO_4_–P (kg/mon)	0.30	0.24	0.36	0.40	0.14	0.12	0.73	0.79	−18.0	0.19
**Validation**										
Flow (l/s)	2.80	2.65	1.45	1.81	2.65	2.32	0.54	0.71	−5.5	0.97
Sediment (t/mon)	0.14	0.14	0.25	0.17	0.06	0.09	0.65	0.67	4.00	0.15
NO_3_–N (kg/mon)	10.63	10.00	11.33	9.08	5.56	7.80	0.70	0.70	−6.10	6.17
NH_4_–N (kg/mon)	0.07	0.08	0.12	0.15	0.03	0.04	0.15	0.53	13.00	0.10
PO_4_–P (kg/mon)	0.41	0.43	0.95	0.64	0.11	0.19	0.66	0.68	5.20	0.55

Uncertainty analysis conducted using SUFI-2 algorithm (Abbaspour et al*.*, 2007), resulted into a p-factor of 0.76, 0.97, and 0.03 for NO_3_–N, PO_4_–P, and NH_4_–N respectively. The r-factor was found to be 1.24, 28.4, and 0.03 for NO_3_–N, PO_4_–P, and NH_4_–N respectively. SUFI-2 predicts model uncertainty using 95PPu, which is the bandwidth between 2.5% and 97.5% levels of cumulative distribution. The p-factor represents the proportion of measured data that falls within the bounds of the 95PPU. The r-factor, on the other hand, is the ratio of the average width of the 95PPU band to the standard deviation of the measured data. A p-factor above 0.7 and an r-factor below 1.5 are indicative of good model performance (Abbaspour et al., 2015; Musyoka et al., 2021). The results show that NH_4_–N had very high uncertainty, with both p and r factors falling outside the acceptable range. Uncertainty analysis results for NO_3_–N were acceptable, while the r-factor for PO_4_–P was outside the recommended range.

**Fig. 4:**
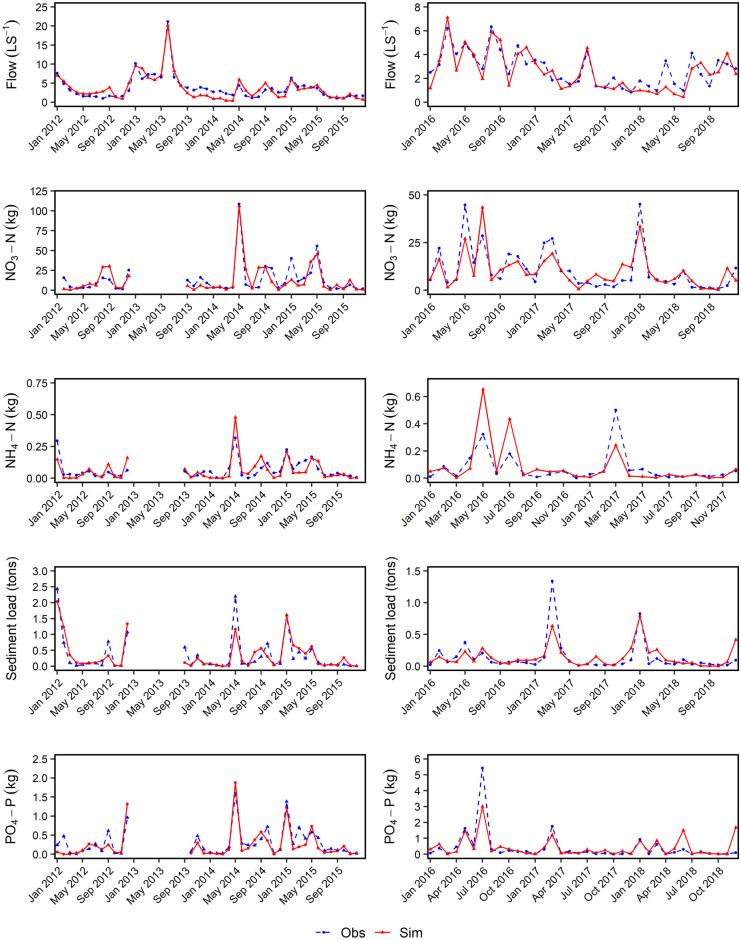
Observed (dashed blue lines) and simulated (red line) hydrographs for each component during model calibration (left) and validation (right)

Time-series plots for calibration and validation also show a good match between simulated and observed data, as shown in Fig. 4

### SLM practice evaluation

The changes in surface runoff, sediment yield, nitrogen, and phosphorus loading at HRU/sub-basin level due to the SLM practices implementation were compared to the BS. Soil loss, runoff, nitrates, and phosphorus loads were calculated at the HRU level for the HRUs where SLM practices were implemented, and the results aggregated to sub-watershed and watershed levels. Paved, forested, and pasture areas were not included in the SLM practices scenario analysis since no SLM practices were implemented on those HRUs. The results were compared at both seasonal and yearly time steps. The effects of the SLM practice implementation on sediment yield, water quality, and quantity were analyzed over a 6-year period (2012–2017). The annual mean runoff, sediment yield, and nutrient loads were calculated for each scenario and evaluated in comparison to BS values during the same simulation period. The load reduction attained was computed by comparing the average annual load of an SLM practice scenario to the baseline scenario.

$$\mathrm{Pecent}\;\mathrm{change}=\frac{\mathrm{postSLM}-\mathrm{BS}}{\mathrm{BS}}\times100$$

where postSLM and BS are runoff, sediment, and nutrient loadings after and before SLM implementation, respectively.

**Fig. 5:**
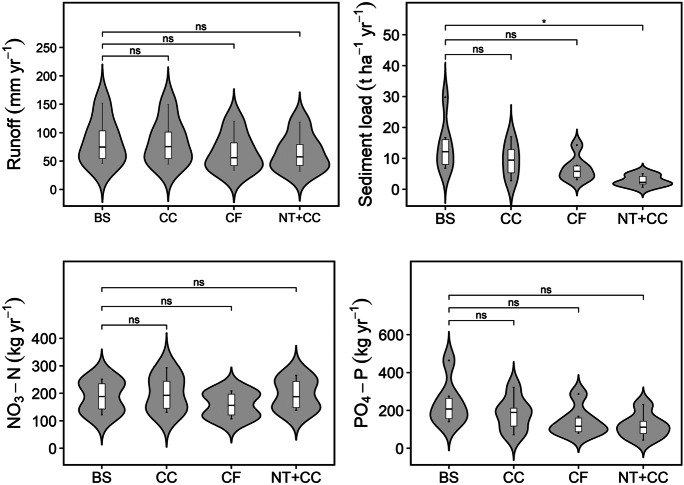
Impact of SLM practices implementation on surface runoff, sediment yield and nutrients over the study period. ns, not significant (*p* > 0.05); *, statistically significant (*p* < 0.05); BS, baseline scenario; CF, contour farming; CC, cover crops; and NT + CC, no till and cover crops

Figure 5 shows the results of each SLM practice implementation on the HOAL catchment. A pairwise Wilcoxon test showed that only sediment yield was significantly (*p* < 0.05) different from the baseline scenario after implementation of NT + CC SLM practice. The other components were not statistically different (*p* > 0.05) from the baseline scenario under all the SLM practices.

All the investigated SLM practices led to decreased runoff from the agricultural fields. The combined no-till and cover crop (NT + CC) SLM practice had a slightly better impact on runoff generation, reducing the amount of surface runoff generated by 19.7 mm (24%) per year over the evaluation period. Contour tillage led to a 23% reduction in surface runoff per year, while cover crops alone led to only a 1% runoff decrease. Contour tillage and no-till practices increase soil surface roughness, leading to reduced flow velocity and consequently decreasing surface runoff due to increased infiltration and improved soil moisture retention. Liu et al. (2013) reported a reduction of surface runoff by 16% and 9% when no-till and contour tillage were implemented in a catchment, respectively. A Wilcoxon pairwise test showed that the annual runoff generated after implementing each of the SLM practices was not statistically different from the calibrated model. Zhao et al. (2001) also reported that tillage had no significant impact on surface tillage in a study conducted in drainage plots in Minnesota River Basin.

**Fig. 6:**
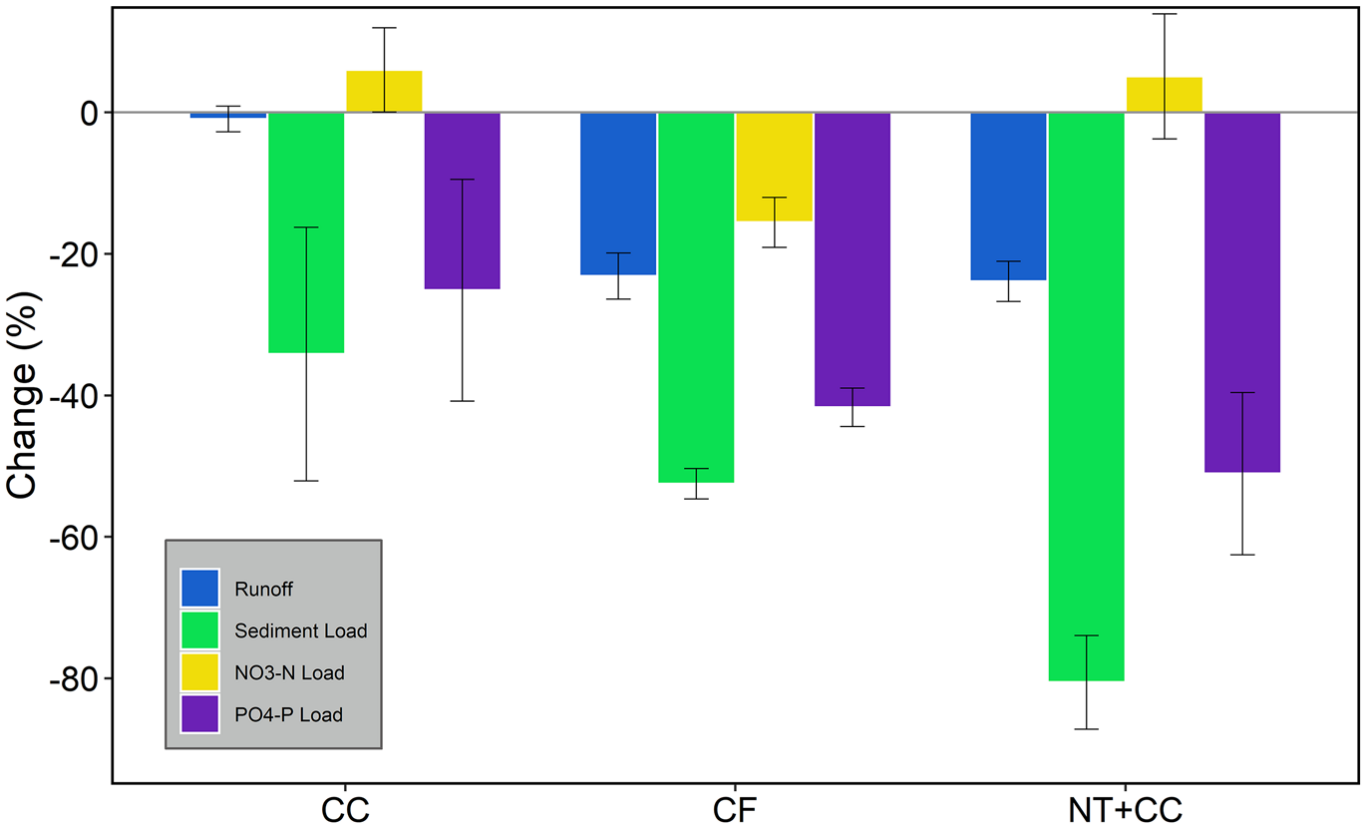
Mean annual reduction in runoff, sediment, and nutrient load (PO_4_–P and NO_3_–N) in the HOAL catchment as a result of implementing SLM practices. CF, contour farming; CC, cover crops; and NT + CC, no-till and cover crops

Sediment yield was the most responsive component to SLM practices implementation, as illustrated in Fig. 6. All SLM practices other than CC (34%) achieved a reduction in the amount of soil loss of more than 50%. With a reduction of 80%, the NT + CC scenario was the most efficient in reducing mean annual sediment loss from the agricultural fields, whereas CF led to a 53% decrease in sediment yield. No-till systems, contour farming, and cover crops can effectively limit soil erosion by lowering the erosive power of runoff and increasing the ability of the land to hold onto the soil (Klik & Rosner, 2020). Similar results were reported by Douglas-Mankin et al. (2013), whereby the no-till practice reduced mean annual sediment load by 72%, whereas contour farming reduced sediment load by 52%. Solieau et al. (1994) also reported a 56% reduction in sediment load when conservation tillage was implemented in a 3.8-ha watershed in northwestern Alabama. Additionally, Tuppad et al. (2010) also reported a 60% reduction in sediment load in the 4282 km^2^ Bosque River Watershed. It is worth noting that rapeseed, winter barley, and winter wheat are cultivated during the winter season in most of the fields, which provides crop cover during this period. Cover crops were therefore only implemented in the SWAT model during seasons where the land was left bare during winter. This explains the low impact of CC on sediment yields.

Concerning nitrogen load, the results indicate that implementation of the selected SLM practices does not have a significant impact. The contour farming practice achieved a NO_3_–N load reduction of 16%, whereas introducing winter cover crops led to an increase in the amount of NO_3_–N load by 6%, and the NT + CC scenario resulted in a 5% increase in NO_3_–N. Other studies (Lam et al., 2011; Wallace et al., 2017) have also found an insignificant impact of SLM practice implementation on nitrogen loads. Additionally, studies by Burgess et al. (1999), Dechmi and Skhiri (2013), Rochette (2008), Rousseau et al. (2012), and Sharpley et al. (2015) also reported increases in NO_3_–N losses after no-till implementation in agricultural fields. Djodjic et al. (2002) attributed increased nutrient loading to increased accumulation of nutrients on the surface resulting from the increasing surface crop residue due to the no-till practice. This residue accumulation leads to high nitrogen mineralization rates (Chen et al., 2014).

Tillage practices impact soil–water movement, which influences denitrification and nitrification processes and also affects water movement through the soil. Returning crop residues into the soil can affectively enhance biological activities, increase phosphorus and nitrogen in the soil, and also improve the soil’s physical properties (Chen et al., 2014). Therefore, nitrogen mobilization and release are influenced by the quality and amount of plant residues in the soil (Burgess et al., 1999). Nitrogen is introduced into the soil through fertilizer application, crop residues, atmospheric deposition, and N_2_ fixation, whereas removal can occur through denitrification, plant uptake leaching, and volatilization (Neitsch et al., 2011). More crop residue on the NT + CC and CC practices can be attributed to the increased NO_3_–N load when cover crops are planted in agricultural fields. Decomposition and mineralization of the fresh organic nitrogen pool can occur in the topsoil layer after the plant residue is left on the ground after harvesting (Liu et al., 2005).

Contrary to our research findings, Motsinger et al. (2016) reported a reduction in nitrate discharge by using rye as a cover crop. Their research findings unveil that the type of crop used as a cover crop could significantly impact the results. Rye is a nitrogen scavenger that was capable of utilizing any excess fertilizers left in the soil after the corn had been removed from the fields, while our study used clovers as cover crops. Similarly, Lam et al. (2011) reported rye as the most efficient crop in minimizing nitrogen losses in a 50-km^2^ lowland catchment in the north of Germany. Quantities of fertilizer application were also another contributing factor to the discrepancies in these studies.

NT + CC reduced PO_4_–P by 51%, CF by 42%, and CC by 25% (Fig. 6). Similar to NO_3_–N, the PO_4_–P load increased with the implementation of both CC and NT + CC. More than 90% of the total phosphorus was transported from the agricultural fields into the stream in the particulate form attached to the soil particles, hence the high correlation between sediment and phosphorus loads.

**Fig. 7:**
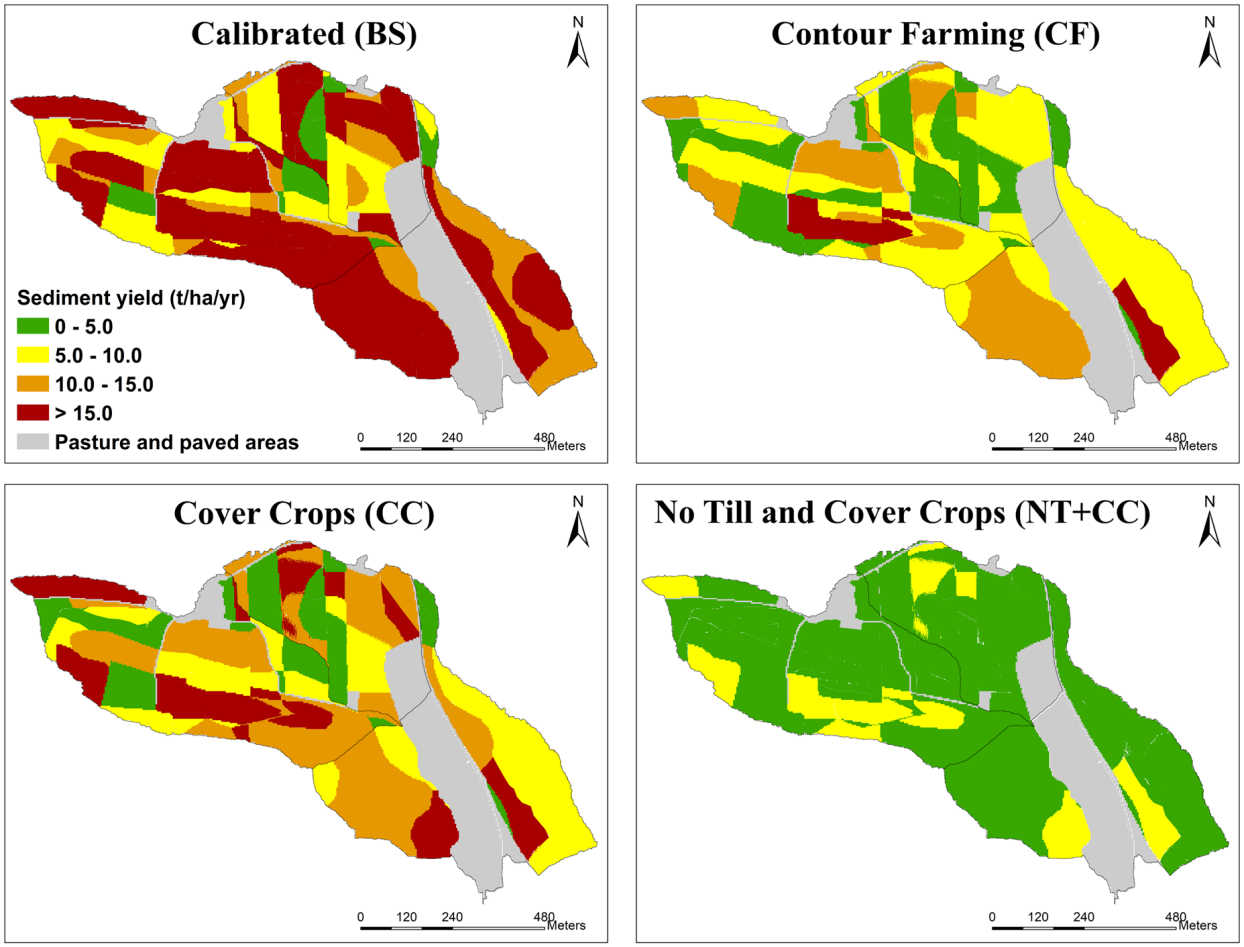
Annual sediment loss from agricultural fields for BS, CF, CC, and NT + CC sustainable land management practices

Figure 7 illustrates the changes in soil loss in the agricultural fields where the studied SLM practices were implemented. The NT + CC SLM practice was able to limit erosion from the fields to 10 tons/ha/year compared to the other SLM practices. In general, all the SLM practices efficiently reduced sediment loads from the HRUs, and sub-catchments compared to the baseline scenario. Winter cover crops as already implemented by some farmers provide a viable solution to soil loss, however, integrating cover crops with no-till given an even more efficient soil conservation practice.

**Fig. 8:**
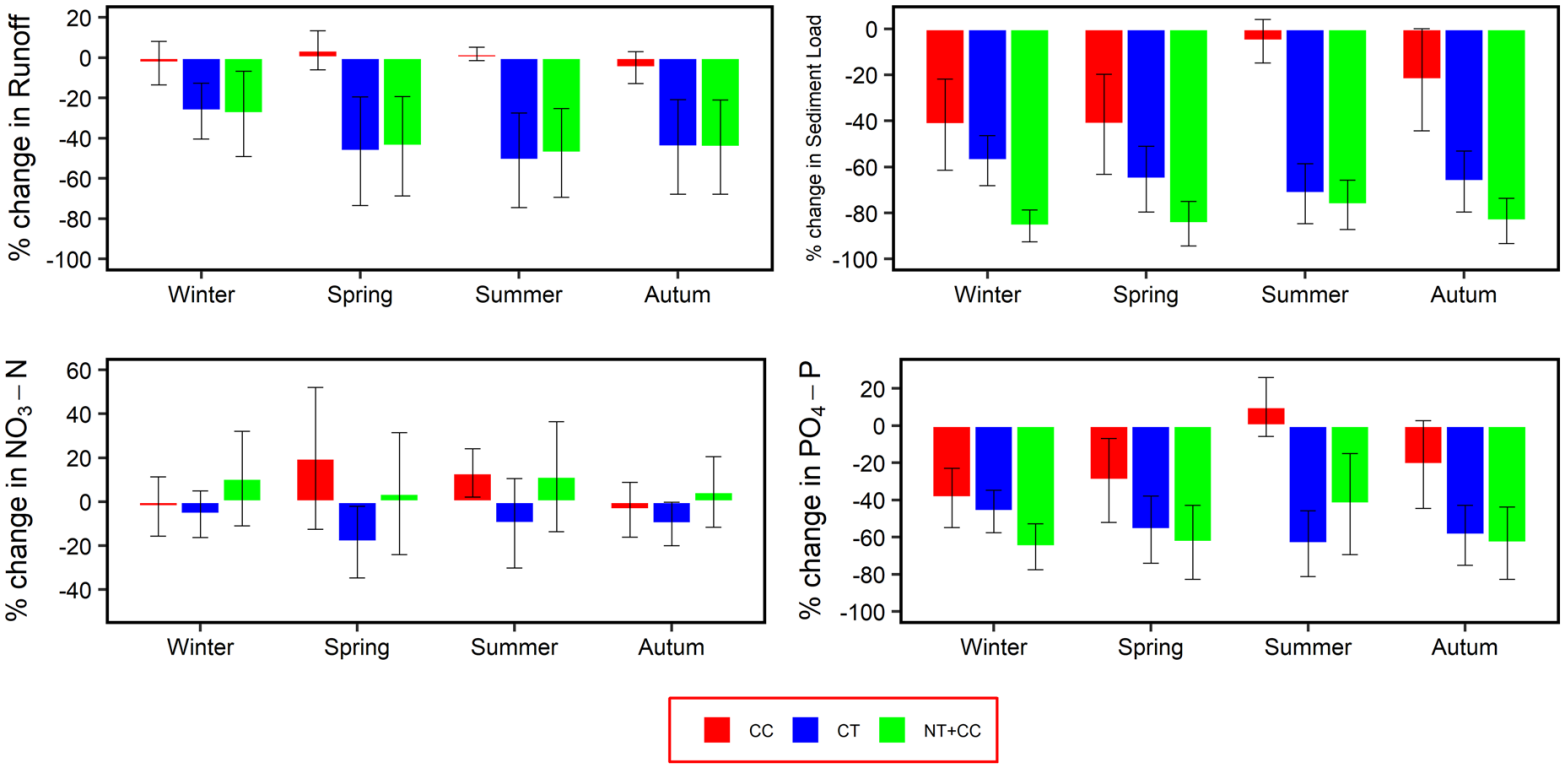
Impact of SLM practices implementation in the HOAL catchment on a seasonal basis

A similar trend was observed on the seasonal time scale, as shown in Fig. 8. NT + CC practice showed better results in reducing sediment loading into the stream in all seasons. NT + CC and CF practices had the same impact on runoff, whereas CC had a minimal impact. NT + CC practice also had a better impact on PO_4_–P in all seasons apart from during summer, while only CF was able to reduce NO_3_–N load in all the seasons. Since cover crops were planted during autumn, winter, and parts of spring, CC had the least impact during the summer period, reducing sediment yield by only 5% compared to autumn (−22%), spring (−41%), and winter (−42%). Cover crops also resulted in increased NO_3_−N loading during spring and summer periods. This increase relates with an accumulation of residues on the soil surface brought about by incorporating dead plant remains into the soil, which led to high nitrogen mineralization rates as discussed earlier.

This study indicates that implementing NT + CC will lead to annual conservation of 750 tons of soil, while CC and CF will lead to 311 and 489 soil loss reductions in tons per year. PO_4_–P input into the streams will be reduced by 120 kg/year by implementing NT + CC, while CF and CC will lead to a reduction in PO_4_–P load by 98 kg/year and 59 kg/year, respectively. Thus, the implementation of SLM practices could have a long-term effect on agricultural farms’ productivity and overall operating costs. When phosphorus and sediments reach freshwater bodies, they cause pollution, and authorities may be required to spend significant sums to clean up the mess created by these pollutants by desilting dams and restoring polluted water bodies. According to the findings of this study, implementing SLM practices at the farm level may alleviate some of these challenges.

Previous studies have demonstrated that implementing SLM practices in agricultural watersheds could have long-term economic benefits. Lam et al. (2011) stressed the importance of the trade-off between SLM practice effectiveness and cost. They applied a cost–benefit analysis method put forward by Gitau et al. (2004) and reported that the increased effectiveness of SLM practice would be complemented by an increase in operating costs, and it is, therefore, necessary to find a balance.

## Conclusion

The aims of this paper were to investigate the impact of selected sustainable land management (SLM) practices on erosion processes and transport of agrochemicals within an agriculturally used catchment, as well as the SLM practices’ ability to mitigate environmental challenges posed by suspended sediments and nutrients when they find their way into water bodies. A SWAT model was set up and subsequently calibrated and validated for stream flow, sediment yield, NO_3_–N, NH_4_–N, and PO_4_–P at the watershed outlet.

The results of this study demonstrated a good correlation between observed and simulated monthly discharges, achieving *R*^2^ and NSE values greater than 0.85 during the calibration period. During the validation period, the NSE and *R*^2^ for flow were 0.54 and 0.71, respectively. Sediment, NO_3_–N, and PO_4_–P were also well simulated with NSE ranging between 0.65 and 0.79 during the calibration and validation periods, whereas NH_4_–N was not well simulated with NSE of 0.47 and 0.15 during calibration and validation, respectively. Overall, the SWAT model performance can be deemed satisfactory in modeling flow, sediment, and nutrient load in the HOAL catchment.

Four selected SLM practice scenarios, which included the baseline scenario (BS), contour farming (CF), cover crops (CC), and a combination of no-till and cover crops (NT + CC), were simulated, and their effectiveness in reducing surface runoff, sediment yield, and nutrients at the HRU and watershed level was assessed. The simulated SLM practices were compared with the calibrated model to examine any changes in the simulated components resulting from SLM practice implementation. The SLM practices were only implemented in the agricultural fields, excluding paved and forested areas. All the simulated SLM practices had a positive impact on the mean annual runoff and sediment load from the agricultural fields. The NT + CC scenario was the most efficient in reducing sediment load from the HRUs, achieving a mean annual reduction of 80% compared to the BS. CF and NT + CC SLM practices achieved similar results in runoff reduction of about 23%, whereas the CC practice resulted in a 1% decrease in surface runoff. Based on the findings of this study, a combination of no-till and cover crops was found to be most effective towards reducing erosion and surface runoff. However, contour farming was more effective in reducing nitrate and PO_4_–P loads.

The findings of this study show that a proper combination of available SLM practices can lead to a significant reduction in soil loss from agricultural fields, which can eventually lead to more sustainable and environmentally friendly agricultural practices at a watershed level. The SWAT model obtained good simulation results for nutrients and sediment and was capable of accurately representing physical processes in the catchment. The modeling results can therefore be used to support adequate soil and water management plans. The present investigation was conducted in a small, homogeneous catchment, where the accessibility of data was not a challenge. Hence, it would be advisable to adopt the same research approach in large, heterogeneous watersheds with limited data availability.

## Supplementary Information

Below is the link to the electronic supplementary material.

Supplementary file1 (ZIP 169 KB)

## Data Availability

Datasets and other materials obtained during this study are available from the corresponding author and may be accessible at any time upon request.
