# Supplementary figures and images for: Evaluating the impacts of sustainable land management practices on water quality in an agricultural catchment in Lower Austria using SWAT
Source: Environ Monit Assess. 2023 Mar 25;195(4):512. doi: 10.1007/s10661-023-11079-y (PMC10039844; doi:10.1007/s10661-023-11079-y)

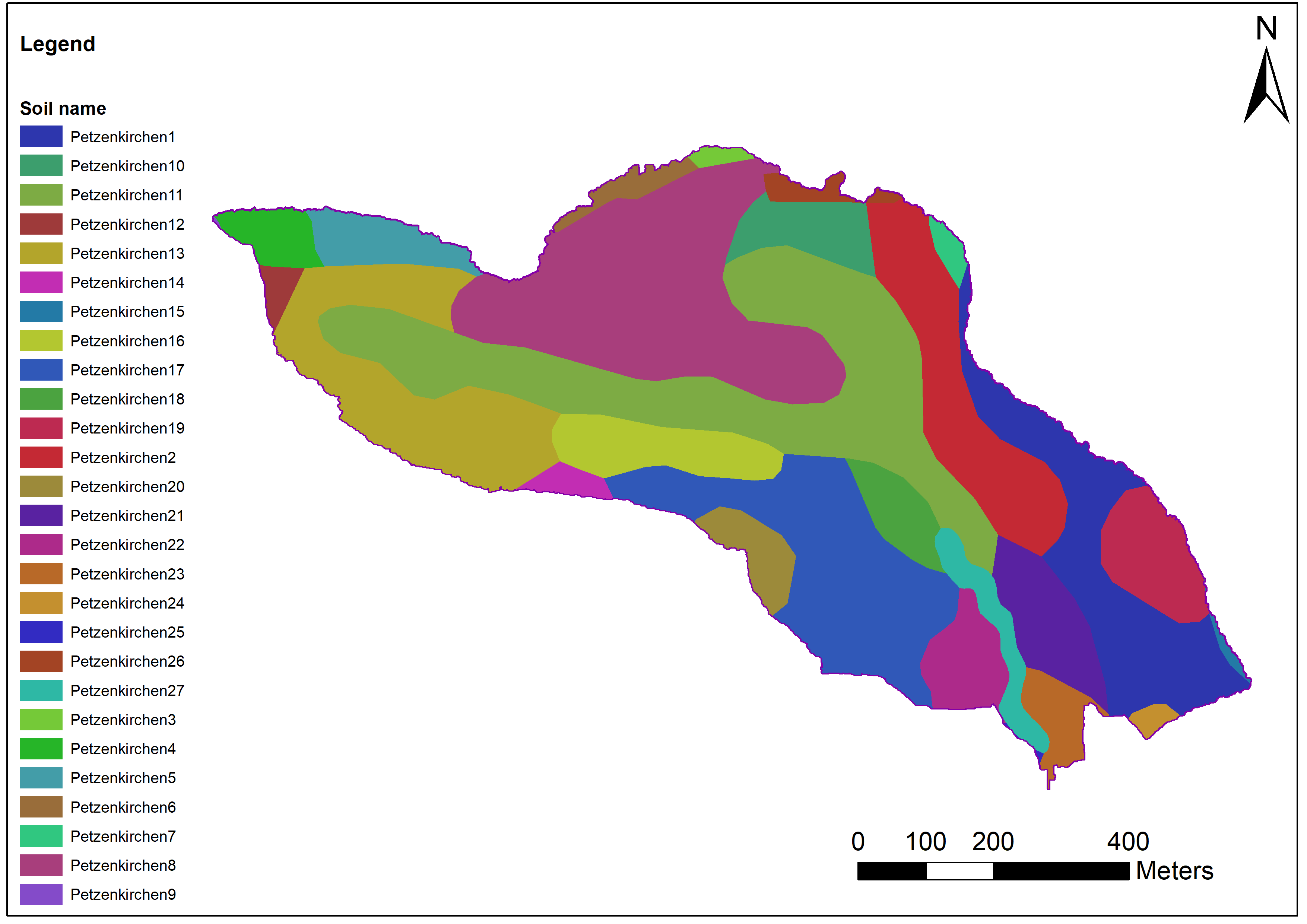

Supplement: Supplementary file 1 — Supplementary file1 (ZIP 169 KB) [file 10661_2023_11079_MOESM1_ESM.zip › Soils.tif]
